# How Does Accounting for Worker Productivity Affect the Measured Cost-Effectiveness of Lumbar Discectomy?

**DOI:** 10.1007/s11999-013-3440-6

**Published:** 2014-01-03

**Authors:** Lane Koenig, Timothy M. Dall, Qian Gu, Josh Saavoss, Michael F. Schafer

**Affiliations:** 1 KNG Health Consulting, LLC, 15245 Shady Grove Road, Suite 305, Rockville, MD 20850 USA; 2 IHS Global, Inc, Washington, DC USA; 3 Econometrica, Inc, Bethesda, MD USA; 4 Department of Orthopaedic Surgery, Northwestern University Feinberg School of Medicine, Chicago, IL USA

## Abstract

**Background:**

Back pain attributable to lumbar disc herniation is a substantial cause of reduced workplace productivity. Disc herniation surgery is effective in reducing pain and improving function. However, few studies have examined the effects of surgery on worker productivity.

**Questions/purposes:**

We wished to determine the effect of disc herniation surgery on workers’ earnings and missed workdays and how accounting for this effect influences the cost-effectiveness of surgery?

**Methods:**

Regression models were estimated using data from the National Health Interview Survey to assess the effects of lower back pain caused by disc herniation on earnings and missed workdays. The results were incorporated into Markov models to compare societal costs associated with surgical and nonsurgical treatments for privately insured, working patients. Clinical outcomes and utilities were based on results from the Spine Patient Outcomes Research Trial and additional clinical literature.

**Results:**

We estimate average annual earnings of $47,619 with surgery and $45,694 with nonsurgical treatment. The increased earnings for patients receiving surgery as compared with nonsurgical treatment is equal to $1925 (95% CI, $1121–$2728). After surgery, we also estimate that workers receiving surgery miss, on average, 3 fewer days per year than if workers had received nonsurgical treatment (95% CI, 2.4–3.7 days). However, these fewer missed work days only partially offset the assumed 20 workdays missed to recover from surgery. More fully accounting for the effects of disc herniation surgery on productivity reduced the cost of surgery per quality-adjusted life year (QALY) from $52,416 to $35,146 using a 4-year time horizon and from $27,359 to $4186 using an 8-year time horizon. According to a sensitivity analysis, the 4-year cost per QALY varies between $27,921 and $49,787 depending on model assumptions.

**Conclusions:**

Increased worker earnings resulting from disc herniation surgery may offset the increased direct medical costs associated with surgery. After accounting for the effects on productivity, disc herniation surgery was found to be a highly cost-effective surgery and may yield net societal savings if the benefits of outpatient and inpatient surgery persist beyond 6 and 12 years, respectively.

**Level of Evidence:**

Level II, economic and decision analysis. See the Instructions for Authors for a complete description of levels of evidence.

## Introduction

With the majority of inpatient disc herniation surgeries performed on working-aged individuals, successful treatment of lumbar disc herniation has the potential to yield substantial benefits in terms of employee productivity [1]. In the 2008 National Health Interview Survey (NHIS) [6], an estimated 10.5 million people reported having back problems with radiating leg pain. On average, this population reported spending 34 days in bed, and missing 26 workdays in the prior 12 months (among respondents with a work history) [24]. In another study, back pain was estimated to cause an average of 5.3 hours of lost productive time at work per week with most of that lost time the result of reduced performance [20].

Surgical treatment of lumbar disc herniation has been shown to be cost-effective. For example, the Spine Patient Outcomes Research Trial (SPORT), a prospective multicenter study, showed improved clinical outcomes (pain, physical function, and disability) for patients who had surgery for lumbar disc herniation relative to nonsurgical treatment [26, 27]. During a 4-year period, lumbar disc herniation was found to be cost-effective in the pooled randomized and observational cohorts at an incremental cost of $43,800 per additional quality-adjusted life-year (QALY) with surgical treatment priced at estimated private-payer levels [23]. The SPORT findings are consistent with those of other studies that have shown that surgery for disc herniation produces better outcomes than nonsurgical treatment [3, 9]. However, prior studies have not taken into account improvements in worker productivity as a result of surgery for lumbar disc herniation, and as such, likely have underestimated the cost-effectiveness of this intervention.

The purpose of our study was to assess the cost-effectiveness of lumbar disc herniation surgery after accounting for its affect on worker productivity. The research addressed two questions. First, we examined the affect of surgery on workers’ productivity using data from SPORT [27] (based on the pooled randomized and observation cohorts analyzed on an as-treated basis) and the NHIS [6]. Second, we assessed how the inclusion of these factors influenced the cost-effectiveness of surgical treatment for lumbar disc herniation.

## Materials and Methods

We used regression modeling and decision analysis to estimate the incremental cost-effectiveness of disc herniation surgery on a working population. The overall approach followed those of Dall et al. [8] and Ruiz et al. [19]. The model was estimated for different age-cohorts (younger than 40 years, 40–44, 45–49, 50–54, 55–59, and 60–64 years), and averages were estimated using a weighted mean based on the age distribution of patients receiving surgery. We included sensitivity analysis to test the model’s robustness to changes in our model assumptions. Further details on methods are provided in Appendix 1.

### Estimating the Effects of Functional Limitations on Earnings and Missed Workdays for Individuals with Back Pain Radiating Down the Leg

To estimate the effects of lumbar disc herniation on worker productivity, we used information on functional limitations, missed workdays, and income from the NHIS [6]. The relationships between functional status and earnings, and between functional status and missed workdays, respectively, were determined by least squares and negative binomial regressions. The analyses were run on a sample of NHIS respondents who reported limitations as a result of back pain that had spread down the leg and below the knee. We used the models to determine workers’ number of missed workdays and household earnings conditional on their level of functional ability in a given year.

The key explanatory variable in the regression models was a functional limitations index score. We obtained predicted values for earnings and missed workdays for the surgical and nonsurgical groups by using the average functional limitation index scores derived from the randomized and observational cohorts in SPORT (across 1, 2, and 4 years posttreatment) and the results of the regression models [27]. We also assumed that workers lost a mean of 20 workdays to recover from lumbar disc herniation surgery based on the Official Disability Guidelines [29]. Missed workdays were converted to a dollar value using average earnings-per-day estimates. These results were incorporated into a Markov model.

### Markov Model

We used a Markov cohort analysis to estimate the differences in direct medical costs, indirect costs, and QALYs between surgical and nonsurgical treatments of lumbar disc herniation. We assumed that patients who are treated nonsurgically do not receive surgical treatment for disc herniation during the model time horizon. We did not distinguish between microdiscectomy and open discectomy in the model because outcomes, in terms of physical health and function, are comparable [25]. Direct medical costs were determined from a private-payer perspective and included patient out-of-pocket expenses. To estimate direct medical costs for surgery, we analyzed Medicare claims data to determine average Medicare payments for disc herniation surgery. These payments then were adjusted to account for differences in private payer and Medicare payment levels. Indirect costs were based on worker productivity and were assessed by changes in earnings and number of missed work days.

**Fig. 1:**
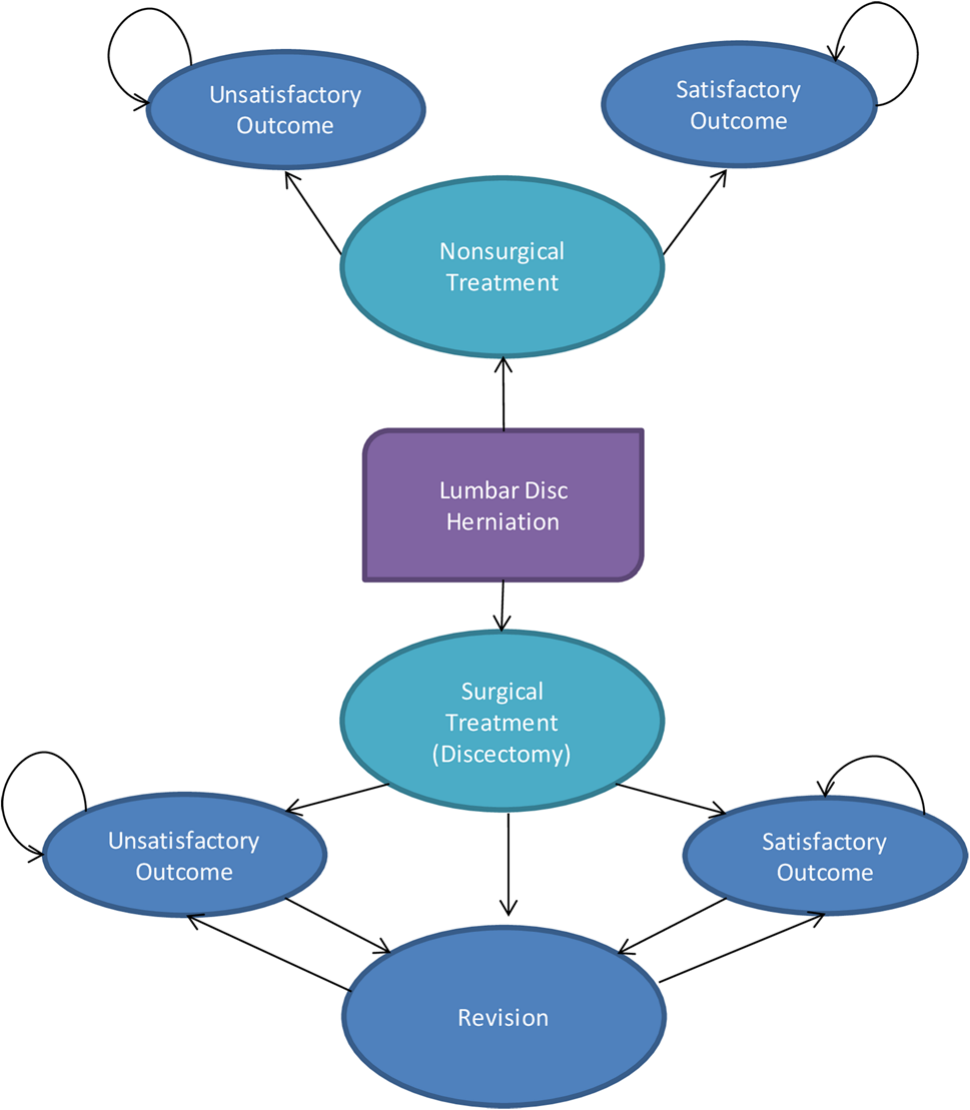
The treatment pathway and health states in the Markov model of lumbar disc herniation are shown. The surgical treatment branch of lumbar disc herniation consists of four health states: “Dead”, “Satisfactory outcome”, “Unsatisfactory outcome” and “Revision”. Within the first year after surgery, alive patients can have “Revision” surgery or they can have either a “Satisfactory outcome” or an “Unsatisfactory outcome”. “Revision” is a temporary health state, meaning alive patients in the “Revision” state will transition to either a “Satisfactory outcome” or an “Unsatisfactory outcome” in the next cycle. For patients in either the “Satisfactory outcome” or “Unsatisfactory outcome” state, they can stay there until they die or they can have “Revision” surgery in subsequent years. The nonsurgical treatment branch of lumbar disc herniation consists of three health states: “Dead”, “Satisfactory outcome”, and “Unsatisfactory outcome”. Within the first year after nonsurgical treatment, alive patients can have either a “Satisfactory outcome” or an “Unsatisfactory outcome”. Once they are in either state, they will stay there until they die. The “Dead” state is not shown.

In our base model, we ran the Markov model for 4 years to correspond to the period of observation in the data obtained from SPORT. However, we also explored additional time horizons. All costs and utilities reflect a 3% annual discount rate in the Markov model. A patient starts in a pretreatment state with lumbar disc herniation and undergoes either surgical (discectomy) or nonsurgical treatment (Fig. 1). Nonsurgically treated patients enter either a satisfactory or unsatisfactory health state where they remain. In the surgical pathway, a patient may die either perioperatively or postoperatively, have a satisfactory outcome, have an unsatisfactory outcome, or have revision surgery during the first year after primary surgery. Patients in satisfactory and unsatisfactory health states may stay there until natural death or have a relapse and have revision surgery, at which point they may either die or achieve a satisfactory or unsatisfactory outcome. Death is an absorbed state in the model. The model was estimated in TreeAge Pro 2011 (TreeAge Software, Inc, Williamstown, MA, USA) using the Markov model transition probability matrix.

### Clinical Parameters

In 2010, a systematic review of randomized clinical trials to assess the effectiveness of surgical treatment for disc herniation was published [13]. This review identified two studies with a low risk of bias. Of these two studies, one included surgical patients in the comparison group, and thus was unsuitable for our purposes [18]. In the other study from SPORT, significant crossover between the treatment and comparison groups compromised the study’s randomization and allowed for potential bias from carryover effects [28].

Given the limitations of randomized clinical studies, we conducted a comprehensive literature review of observational studies that compared surgical treatment for disc herniation with conservative therapy. We identified two studies that met our inclusion criteria by having prospectively collected data, a nonsurgical treatment comparison group, statistical adjustment for baseline differences between the surgical and nonsurgical groups, and a large sample size [4, 28]. Of these, the SPORT study [28] that combined the randomized and observational cohorts into an as-treated analysis was the largest and more recent study. The measured probability of a satisfactory outcome, revision rates, surgical mortality, and utilities were largely consistent between these two observational studies. Importantly, we were able to obtain information regarding comparative functional status from the SPORT pooled cohort to estimate the effects of disc herniation surgery on earnings and missed workdays. Basing our clinical assumptions on the same study offered advantages of consistency. For these reasons we chose to, when possible, use the findings from the SPORT study to populate the model’s probabilities, although sensitivity analysis was conducted on these assumptions (Table 1).

**Table 1:** Parameters and utilities in base Markov model

Variable	Surgical treatment	Nonsurgical treatment
Clinical parameter		
Fraction of patients with satisfactory outcome after treatment	0.753 [26, 27]	0.485 [26, 27]
Fraction of patients with satisfactory outcome after revision	0.753 [26, 27]	NA
Annual revision rate after surgery–first year	0.06 [26]	NA
Annual revision rate after surgery–subsequent years	0.03 [4, 16, 26]	NA
Surgical mortality rate	0.0013 [26]	NA
Natural mortality rate	US life table	US life table
Utility		
Dead	0	0
Satisfactory outcome	0.89 [14]	0.89 [14]
Unsatisfactory outcome	0.56 [14]	0.56 [14]
Revision surgery	0.69 [14, 26, 27]	NA

NA = not applicable.

We defined success as patients’ satisfaction with the results after treatment. Weinstein et al. [27] reported that 75% of patients treated with surgery and 48.8% of patients treated nonsurgically indicated they were “very/somewhat satisfied with symptoms” at 2 years after treatment. The percentage did not change significantly at 3 and 4 years after treatment for both treatment groups (ie, 74.3% and 76.6% for the surgery group and 49.9% and 46.7% for the nonsurgery group) [27]. Among participants of the Maine Lumbar Spine Study [4], 71% of patients treated with surgery and 56% of patients with nonsurgical treatment indicated they were satisfied with their current state at 10 years followup. In our model, we set the percentages of patients with satisfactory outcomes at 75.3% for the surgery group and 48.5% for the nonsurgery group, which are the averages across outcomes at 2, 3, and 4 years after treatment based on SPORT data. These success rates of surgical treatment of lumbar disc herniation are consistent with the ranges reported in the literature of 70% to greater than 90% [11]. The percent of patients with satisfactory outcomes after the revision surgery was set to be the same as after the initial surgery (ie, 75.3%) because studies have reported comparable outcomes after revision surgery and initial surgery for lumbar disc herniation [17, 21].

SPORT data suggested that the revision rate in the first year after surgery is higher than in subsequent years [27]. Specifically, the weighted cumulative revision rates combining the randomized and observational cohorts were 6% 1 year after surgery, 8% 2 years after surgery, 9% 3 years after surgery, and 10% 4 years after surgery. This translates to an approximate annual revision rate of 2.5%. Data from the Maine Lumbar Spine Study [4] revealed that 64 of the 217 patients (ie, 29%) treated with surgery and who were still alive by the 10-year followup had a reoperation. Osterman et al. [16] reported that, among patients with one revision after lumbar discectomy, 25.1% experienced additional spinal surgery before the 10-year followup, which indicated an approximate annual second reoperation rate of 2.5%. In our model, the first-year revision rate was set at 6% and the annual reoperation rate in the subsequent years was set at 3% per year. Patients were allowed to have up to two revisions in the Markov model. Surgical mortality was set to 0.14% [27]. Natural mortality comes from the age- and sex-specific mortality in the US life tables [2].

Two cost-effectiveness studies using SPORT data reported the average EuroQol-5 dimensions (EQ-5D) posttreatment utility level of patients treated either surgically or nonsurgically [22, 23]. The average utility of patients after treatment was approximately 0.8 for surgical treatment and 0.7 for nonsurgical treatment (where utility of 1.0 indicates no decline in quality of life associated with health problems and utility of 0.8 indicates a 20% decline in quality of life). However, neither study reported the average utility separately for patients satisfied and dissatisfied with the symptoms. Based on data from the Beaver Dam Health Outcome Study, Malter et al. [14] reported a time tradeoff utility level of 0.89 for patients with satisfactory outcomes and 0.56 for patients with unsatisfactory outcomes after treatment of lumbar disc herniation. Using these utility values and success rates of 75.3% and 48.5% for surgical and nonsurgical treatments, respectively, the estimated utility is 0.81 for patients treated with surgery and 0.72 for patients treated nonsurgically, which is consistent with the EQ-5D utility from SPORT. We used the utility values reported by Malter et al. [14] in the Markov model (0.89 and 0.56 for satisfactory and unsatisfactory outcomes, respectively). The utility value of the temporary revision state was set at 0.69 (the midpoint between utility of surgery treatment and utility of unsatisfactory outcomes) until the revision reached full benefit in the next cycle. A utility of zero was assigned to the dead state.

We used the 2009 5% Medicare claims [7] to estimate the surgery payments and select 2009 State Ambulatory Surgery Databases [12] (Colorado, New Jersey, Florida, and Wisconsin) to estimate the percent of surgeries done in inpatient and outpatient surgical settings. For both settings, surgery cost includes payments to facilities and physicians.

Annual medical costs other than surgery were estimated using medical costs of SPORT participants for 2 years after initial treatment, which include costs associated with healthcare visits, diagnostic tests, medications, and other healthcare services [22]. All cost estimates (Table 2) were adjusted to 2009 dollars and to reflect private-payer reimbursement rates.

**Table 2:** Direct medical cost estimates of treatment of lumbar disc herniation

Type of cost	Direct medical cost
Surgery cost	$16,423
Inpatient only	$20,585
Outpatient only	$11,616
Annual medical costs of patients treated surgically (excluding surgery cost)	$3208 [22]
Annual medical costs of patients treated nonsurgically	$3794 [22]

For the sensitivity analysis, we used at least a 10% range around the base estimates (ie, 10% below and above the base estimates) or ranges suggested in the literature for most parameters [11]. For missed work and earnings, we used lower and upper bounds based on a 95% CI.

## Results

Surgical treatment for disc herniation increases earnings by a mean of $7154 (95% CI, $4166– $10,142) and results in 8.9 additional missed work days (95% CI, 6.3–11.3) during a 4-year period. In total, changes in earnings and missed work days produced a net surgical cost offset equal to $5603. Average annual earnings for surgical and nonsurgical patients are estimated to be $47,619 and $45,694, respectively. Surgical patients receive an annual earnings premium of $1925 (95% CI, $1121–$2788) (Table 3). Surgical patients miss an average of 7.6 work days each year after surgery compared with 10.6 missed days for nonsurgical patients, resulting in 3 fewer missed work days (95% CI, 2.35–3.69) for surgical patients per year. The net present value of this benefit after 4 years is equal to 11.1 fewer missed work days (95% CI, 8.7–13.7). This benefit fails to offset the assumed 20 additional missed work days that occur in the recovery period immediately after surgery. Assuming the value of a work day to be equal to 1/240th of the baseline salary, we estimate that additional missed work days increase the cost of surgery by $1572 (95% CI, $1107–$1983) during a 4-year period. If, as some literature suggests [4, 15], the productivity benefits of surgery persist longer to, for example, 8 years, then during that period, surgical patients would earn $13,510 more than nonsurgical patients (95% CI, $7868–$19,153), and experience approximately the same number of missed work days, implying a total cost offset of $13,664.

**Table 3:** Estimates of functional limitations index scores, household earnings, and value of missed workdays by age group

Age group (years)	Percentage of patients	Average functional limitations score (SPORT)			Household earnings			Value of missed workdays (excludes surgery recovery)		
		Surgical	Nonsurgical	Change	Surgical	Nonsurgical	Change	Surgical	Nonsurgical	Change
< 40	32%	0.86	0.77	0.09	$44,713	$42,903	$1810	$1367	$1869	$503
40–44	15%	0.85	0.75	0.10	$50,524	$48,714	$1810	$1403	$1917	$514
45–49	17%	0.85	0.75	0.10	$49,251	$47,441	$1810	$1509	$2063	$553
50–54	15%	0.87	0.74	0.12	$48,973	$46,741	$2232	$1062	$1561	$499
55–59	12%	0.87	0.74	0.12	$48,803	$46,571	$2232	$1263	$1857	$593
60–64	9%	0.86	0.76	0.10	$46,289	$44,479	$1810	$1358	$1855	$497
Weighted average		0.86	0.76	0.10	$47,619	$45,694	$1925	$1337	$1859	$523

SPORT = Spine Patient Outcomes Research Trial.

Consideration of indirect costs results in the incremental cost-effectiveness ratio of surgery for disc herniation to decrease from $52,416 to $35,146 if the benefit persists during a 4-year period (Table 4). This represents a 34% improvement in cost-effectiveness. During the 4-year period, surgical patients incur 3.04 QALYs and $30,900 in direct medical costs, while nonsurgical patients incur 2.73 QALYs and $14,402 in direct medical costs. Thus, surgical treatment increases direct medical costs by $16,498, while improving QALYs by 0.31. Earnings and missed work days reduce the added cost of surgery by $5603. If productivity benefits from surgery persist to 8 years, the direct costs of surgical and nonsurgical treatments increase to $43,036 and $26,904 respectively, while the QALYs incurred increase to 5.69 and 5.10. After factoring in productivity offsets, this implies an incremental cost-effectiveness ratio of $4186 during an 8-year horizon.

**Table 4:** Costs and additional QALYs from surgical treatment of lumbar disc herniation (4-year time horizon)

Age category	Surgical Treatment					Nonsurgical treatment		ICER [(D–F)/(E–G)]
	Total direct cost (A)	Earnings offsets (B)	Value of missed workday offsets (C)	Net costs (D = A–B–C)	QALY (E)	Total direct cost (F)	QALY (G)	
Overall	$30,900	$7251	($1648)	$25,297	3.04	$14,402	2.73	$35,146
Inpatient	$35,636	$7251	($1648)	$30,033	3.04	$14,402	2.73	$50,423
Outpatient	$25,406	$7251	($1648)	$19,803	3.04	$14,402	2.73	$17,423
Younger than 40 years	$30,979	$6728	($1657)	$25,908	3.07	$14,488	2.75	$35,689
40–44 years	$30,943	$6728	($1856)	$26,071	3.06	$14,489	2.74	$36,194
45–49 years	$30,905	$6728	($1605)	$25,782	3.05	$14,408	2.73	$35,544
50–54 years	$30,851	$8298	($1729)	$24,282	3.03	$14,349	2.72	$32,041
55–59 years	$30,781	$8298	($1363)	$23,846	3.01	$14,274	2.7	$30,878
60–64 years	$30,672	$6728	($1596)	$25,540	2.99	$14,157	2.68	$36,718

QALY = quality-adjusted life-year; ICER = incremental cost-effectiveness ratio.

**Fig. 2:**
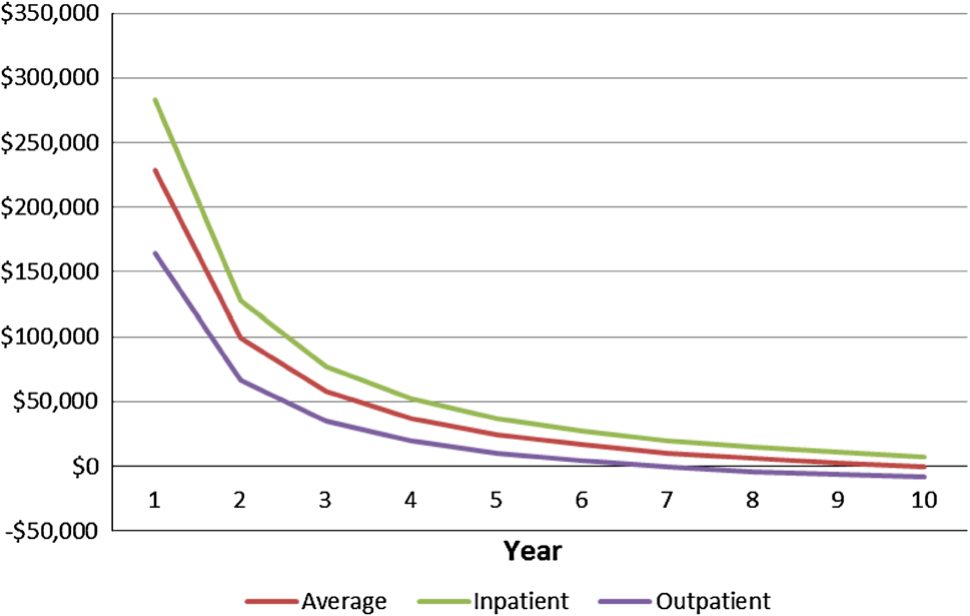
The incremental cost-effectiveness ratios for disc herniation surgery by year of benefit are shown.

We conducted a sensitivity analysis to test the robustness of our findings to modifications in our modeling assumptions based on a 40-year-old patient, which is the approximate average age of patients having a discectomy (Table 5). Our findings are most sensitive to the utility assumptions, and the probabilities of having either a satisfactory or unsatisfactory outcome. For instance, a 10% reduction in the assumed utility of a satisfactory outcome reduces the cost-effectiveness of surgery by 35% (from an incremental cost-effectiveness ratio of $35,861 to $49,787), while a 10% increase improves the cost-effectiveness of surgery by 21% (from $35,861 to $29,263). Varying the earnings effect over its estimated 95% CIs had a much larger effect on the incremental cost-effectiveness ratio ($45,746 to $27,921) than varying the missed work effect over its 95% CI ($38,136, $35,421). Overall, the incremental cost-effectiveness ratio of surgery ranged from $49,987 to $27,921 during the 4-year period in our sensitivity analysis. We also tested the sensitivity of our findings to variations in surgery setting and in duration of the surgical benefit. If the surgery is performed in an inpatient setting, the incremental cost-effectiveness ratio would increase to $50,423, while the incremental cost-effectiveness ratio decreases to $17,423 if performed in an outpatient setting (Table 4; Fig. 2). Cost-effectiveness increases if the benefit from surgery persists for a longer period. The surgery generates cost savings if the benefit persists for at least 10 years.

**Table 5:** Sensitivity analysis of key parameter assumptions (based on 40-year-old patient)

Parameter	Value in base model	Value range tested	Incremental cost effectiveness range
First year revision rate after surgical treatment	0.06	0.04–0.08	$35,583–$38,159
Revision rate in subsequent years after surgical treatment	0.03	0.01–0.05	$33,389–$40,385
Fraction of surgical patients with satisfactory outcome	0.753	0.703–0.803	$45,592–$30,937
Fraction of non-surgical patients with satisfactory outcome	0.485	0.435–0.535	$30,736–$46,025
Utility of satisfactory outcome	0.89	0.80–0.98	$49,787–$29,263
Utility of unsatisfactory outcome	0.56	0.50–0.62	$30,692–$46,134
Annual increase in earnings after surgical treatment, compared with nonsurgical treatment	$1810	$1054–$2566	$45,746–$27,921
Annual reduction in missed workdays after surgical treatment, compared with nonsurgical treatment	2.7	2.2–3.4	$38,136–$35,421
Surgery cost	$16,423	$14,781–$18,065	$30,916–$36,858
Annual medical spending after surgery for disc herniation	$3208	$2887–$3529	$34,151–$39,573
Annual medical spending after nonsurgical treatment for disc herniation	$3794	$3415–$4177	$41,435–$32,232
Missed workdays recovering from disc herniation surgery	20	10–30	$31,031–$42,806

QALY = quality-adjusted life year.

## Discussion

Effective treatment for disc herniation could yield benefits to individuals and employers. Although research has shown benefits associated with surgery for disc herniation [3, 4, 27], neither the SPORT nor the Maine Lumbar Spine Study found statistically significant differences in the probability of being employed between patients receiving surgery and patients treated nonsurgically. We found that during a 4-year period, surgical treatment for disc herniation increased earnings by $7154 and increased missed work days by 8.9 days. In total, the net effect of changes in earnings and missed work days resulted in a surgical cost offset of $5603. The inclusion of indirect costs improved the incremental cost-effectiveness ratio of surgery for disc herniation by 34% from $52,416 to $35,146.

This study has several limitations. First, our estimates of indirect costs are based on a representative population of people with back pain that had spread to the leg and below the knee. We inferred the effects of discectomy on productivity by linking back pain, functional limitations, and earnings. Thus, the reliability of these findings is sensitive to the validity of the model’s assumptions. We included a sensitivity analysis to test the robustness of our findings, but it is impossible to eliminate all uncertainty in a study of this nature. Further research is needed to understand the extent to which our model accurately reflects the relationship between functional limitations and earnings for patients who undergo disc herniation surgery. Second, statistical error was present in the estimation of the relationship between treatment approach and functional status, and the estimation of the relationship between functional status and economic outcomes. Although we have provided confidence intervals for each estimation stage, owing to data limitations, we are unable to provide a measure for how this joint uncertainty affects our final results. Third, the NHIS patient sample was limited to patients with back pain with radiating leg pain. Although these are characteristic symptoms of disc herniation, our sample might have included some patients with other conditions. We assume that the relationship effect of back pain on economic outcomes is independent of the cause of the back pain. Fourth, our information for clinical outcomes differences came from an observational study. Although the results were risk-adjusted, there may be remaining differences in baseline health status that bias the findings. Finally, reimbursement for disc herniation surgery varies among payers and insurance markets. Our estimated costs of surgery to payers and patients are based on average payments, and thus might not always be reflective of costs in all circumstances.

Significant geographic variation exists in the rates of back surgery [5]. This suggests that in some areas, discectomy may be either under- or overused. Our estimates of benefits from discectomy are based on average indirect cost reductions for patients who underwent the procedure, but not all patients are equally good candidates for surgery. Careful consideration of individual patient needs and alternatives to surgical treatment may further increase the societal value of lumbar disc herniation surgery.

Our study showed that functional limitations resulting from lumbar disc herniation were associated with lower earnings and an increased number of missed workdays. The level of improvement in functioning for patients undergoing disc herniation surgery suggests material offsets to the cost of surgery in terms of higher earnings and fewer missed workdays. After incorporating estimates of the effects of surgery on earnings and missed workdays, we found that patients treated surgically gain an additional 0.31 QALYs during a 4-year period at an additional cost of $10,895. We believe that this is the first study to estimate the effects of disc herniation surgery on productivity as captured in earnings. Earnings can be affected by disc herniation in numerous ways. First, employees may not be able to work as many hours (and may need to work part-time instead of full-time). Second, the human capital approach postulates that wages are set equal to a worker’s marginal product. Thus, less productive workers are paid less. Prior research has focused on the effects of disc herniation surgery on employment without consideration for the effect of hours worked or productivity changes on earnings.

These findings imply an incremental cost-effectiveness ratio of $35,146 per QALY gained. By comparison, Tosteson et al. [23] reported an incremental cost-effectiveness ratio of $43,800 per additional QALY for surgery priced at private-payer amounts. The difference between our incremental cost-effectiveness ratio and that reported by Tosteson et al. is explained primarily by our inclusion of offsets from increased earnings.

Disc herniation surgery is measured as more cost-effective when the benefits of surgery in terms of earnings and missed workdays are factored in and when the procedure is performed on an outpatient basis. The value of discectomy may be further enhanced by shifting more clinically appropriate patients to an outpatient setting.

## Appendix 1. Cost-Effectiveness of Lumbar Disc Herniation Surgery in a Working Population

We used data from the National Health Interview Survey (NHIS) [6] to generate regression coefficients that described the statistical relationship between physical functional status and economic outcomes. We applied these coefficients to surgical and nonsurgical outcomes data from the Spine Patient Outcomes Research Trial (SPORT) [27] to estimate the effect of surgery on income, missed workdays, and the probability of receiving disability payments. These findings were inputted in a Markov decision model to estimate total societal savings resulting from surgical treatment of lumbar disc herniation.

### Functional Limitation Index Score from the SPORT Data

From SPORT, we obtained average functional limitation index scores based on select questions from the SF-36 and Oswestry instrument that correspond to those in the NHIS. Baseline and postsurgical index scores were obtained for the surgical and nonsurgical groups by five age categories (18–29, 30–39, 40–49, 50–59, and 60–69 years).

The functional limitation index incorporated responses from the following questions:

- Does your health limit you in walking several blocks? (SF-36)
- Does your health limit you in climbing one flight of stairs? (SF-36)
- Does your health limit you in bending, kneeling, or stooping? (SF-36)
- Does your health limit you in lifting or carrying groceries? (SF-36)
- Does your health limit you in moderate activities, such as moving a table, pushing a vacuum cleaner, bowling, or playing golf? (SF-36)
- How has pain affected your ability to sit? (Oswestry)
- How has pain affected your ability to stand? (Oswestry)

An algorithm was used to encode each response into a point value of 1 (severely affected), 2 (moderately affected), or 3 (not affected). The responses were mapped in accordance with Tables 6 and 7 in this appendix. The index point value for each individual was determined by calculating the sum of the corresponding value to each response. This total then was divided by the maximum score of 21 to produce a given patient’s custom index score.

**Table 6:** Conversion values for functional limitation index and SF-36

Index point value	SF-36 response
1 – Severely affected	1 – Limited a lot
2 – Moderately affected	2 – Limited a little
3 – Not affected	3 – Not limited at all

**Table 7:** Conversion values for functional limitation index and Oswestry

Index point value	Oswestry response
1 – Severely affected	2 – Pain prevents me from sitting/standing for more than 1 hour
	3 – Pain prevents me from sitting/standing for more than ½ hour
	4 – Pain prevents me from sitting/standing for more than 10 minutes
	5 – Pain prevents me from sitting/standing at all
2 – Moderately affected	1 – I can sit/stand as long as I like with pain or special accommodations
3 – Not affected	0 – I can sit/stand as long as I like

To validate that our index based on SF-36 and Oswestry information would yield similar index scores to the NHIS questions, we collected patient outcomes data from a sample of patients with lumbar disc herniation treated with surgery at the Rothman Institute, a physician group practice with multiple locations in the northeast. A total of 54 responses were received. We found that the average functional limitation index scores based on SPORT data were comparable to the data collected at the Rothman Institute.

### Estimating the Relationship Between Functional Limitations and Indirect Costs

The NHIS collects information from a stratified random sample of the US population on physical function, economic factors such as employment status and income, and other patient characteristics. Our analysis combined the 2003 through 2009 National Health Interview Survey (NHIS) files to increase the sample size. We limited the regression analysis to patients with back pain. For functional limitations, the NHIS asks respondents: By yourself, and without using any special equipment, how difficult is it for you to…

- walk a quarter of a mile - about 3 city blocks?
- walk up 10 steps without resting?
- sit for about 2 hours?
- reach up over your head?
- stand or be on your feet for about 2 hours?
- stoop, bend, or kneel?
- lift or carry something as heavy as 10 pounds such as a full bag of groceries?
- push or pull large objects like a living room chair?

Responses to each question include: (1) Not at all difficult, (2) Only a little difficult, (3) Somewhat difficult, (4) Very difficult, (5) Can’t do at all. Our analysis focuses only on functional limitations where the person indicates that back pain contributed to his or her limitations.

Using responses from the NHIS sample, we computed a functional limitation index score as described above and the index point values as specified in Table 8 in this appendix.

**Table 8:** Conversion values for functional limitation index and NHIS

Index point value	NHIS response
1 – Severely affected	Unable, Very difficult
2 – Moderately affected	Somewhat difficult, Little difficulty
3 – Not affected	Not difficult

Using regression analysis we compared outcomes for adults with more functional limitations with economic outcomes for adults with fewer functional limitations—controlling for age group (18–39, 40–44, 45–49, 50–54, 55–59, 60 to 64, 65–69, and 70 years and older), sex, highest education attainment (high school diploma, baccalaureate degree, postbaccalaureate degree), and occupation (for analysis of the employed population).

Ordinary least squares and negative binomial regressions were used to quantify the affect of functional limitations on household income for the employed population and missed workdays, respectively.

### Regression Results from National Health Interview Survey (NHIS)

The key explanatory variable in the regression models was the functional limitations index score. We derived this score using responses to NHIS questions and then compared outcomes for adults with different functional limitations scores controlling for other covariates. Model results are shown in Table 9.

**Table 9:** Regression models

Variable	Income (Model 1)	Missed work days (Model 2)
Intercept	12118	4.1509***
Male	3608.941**	0.0376
Age group (reference group = age < 40 years)		
40–44 years	5931.52***	−0.1283
45–49 years	4658.094***	−0.0266
50–54 years	4079.044**	−0.3046***
55–59 years	3908.744*	−0.1269
60–64 years	1576.107	−0.0517
Functional limitation index score	18101***	−3.1197***
Difficulty reaching (reference group = no difficulty)		
Only a little difficult	−1336.45	−0.1329
Somewhat difficult	−1988.13	0.0221
Very difficult	−3992.41	−0.0823
Can’t do at all	−4366.71	0.4011
Has mobility difficulty owing to		
Joint injury	−1106.67	0.3522***
Musculoskeletal condition	−2732.06	−0.2745**
Arthritis	657.2187	−0.0044
Highest educational attainment (reference = less than high school)		
High school degree	20426***	0.0147
College (baccalaureate) degree	43601***	0.1033
Postbaccalaureate degree	54301***	−0.2899*
Sample size	2258	2592

Model 1 estimated using ordinary least squares; Model 2 estimated using negative binomial; * <0.10 **<0.05 ***<0.01

### Methods for Combining Indirect Cost Components and SPORT Data

The relationships between functional limitations and indirect costs were determined by least squares and negative binomial regression models. The results from the models allowed us to determine an individual’s number of missed work days, and household income, conditioned on the probability of being employed.

The cost associated with missed days at work in a given year was computed as:

$$ {\text{Productivity loss}} = {\text{estimated baseline income for employed}}\;*\;\left( {{\text{missed work days}}/ 2 40} \right) $$

Additionally, we assumed that workers lost an average of 20 working days (28 calendar days) as a result of the lumbar disc herniation surgery [29].

### Converting Medicare Costs to All-Payer Costs

Cost estimates based on Medicare payment rates will underestimate payments made by private insurers. To reconcile this difference, we adjusted our estimates of direct medical costs using payment rates of private insurers as a percentage of the Medicare rate (as reported in the literature [10]) and then weighted by the national distribution of payers for treatment of lumbar disc herniation. Ginsburg [10] estimated that private insurers, on average, paid 139% of the Medicare payment rates for inpatient care nationally in 2008. He also reported that private insurer payments as a percentage of Medicare rates for outpatient services in selected areas, ranging from 193% in Cleveland to 368% in San Francisco. We used the median of the reported range, which is 280%, to adjust costs of outpatient services. The Medicare Payment Advisory Committee estimated that the private rate for physician services was, on average, 123% of the Medicare rate across all services and areas in 2003 [15]. For all other patients, including those receiving workers’ compensation, we assumed their rate was the same as the average rates of Medicare and private insurers.

## References

[1] Agency for Healthcare Research and Quality. Healthcare Cost and Utilization Project (HCUP). Available at: http://hcupnet.ahrq.gov/HCUPnet.jsp. Accessed October 23, 2013. 21413206

[2] Arias E (2011). United States life tables, 2007. Natl Vital Stat Rep..

[3] Atlas SJ, Keller RB, Chang Y, Deyo RA, Singer DE (2001). Surgical and nonsurgical management of sciatica secondary to a lumbar disc herniation: five-year outcomes from the Maine Lumbar Spine Study. Spine (Phila Pa 1976).

[4] Atlas SJ, Keller RB, Wu YA, Deyo RA, Singer DE (2005). Long-term outcomes of surgical and nonsurgical management of sciatica secondary to a lumbar disc herniation: 10 year results from the Maine lumbar spine study. Spine (Phila Pa 1976).

[5] Center for the Evaluative Clinical Sciences. Preference-sensitive care. *The Dartmouth Atlas of Health Care*. January 15, 2007. Available at: http://www.dartmouthatlas.org/downloads/reports/preference_sensitive.pdf. Accessed December 2, 2013.

[6] Centers for Disease Control and Prevention. National Health Interview Survey. Available at: http://www.cdc.gov/nchs/nhis.htm. Accessed October 23, 2013.

[7] Centers for Medicare & Medicaid Services. MEDPAR Limited Data Set (LDS) - Hospital (National). Available at: http://cms.gov/Research-Statistics-Data-and-Systems/Files-for-Order/LimitedDataSets/MEDPARLDSHospitalNational.html. Accessed January 29, 2013.

[8] Dall TM, Gallo P, Koenig L, Gu Q, Ruiz D (2013). Modeling the indirect economic implications of musculoskeletal disorders and treatment. Cost Eff Resour Alloc..

[9] Gibson JN, Waddell G (2007). Surgical interventions for lumbar disc prolapse: updated Cochrane Review. Spine (Phila Pa 1976).

[10] Ginsburg PB. Wide variation in hospital and physician payment rates evidence of provider market power. HSC Research Brief No. 16. Washington, DC: Center for Studying Health System Change; November 2010. 21117341

[11] Häkkinen A, Kautiainen H, Järvenpää S, Arkela-Kautiainen M, Ylinen J (2007). Changes in the total Oswestry Index and its ten items in females and males pre- and post-surgery for lumbar disc herniation: a 1-year follow-up. Eur Spine J..

[12] Healthcare Cost and Utilization Project (HCUP). Overview of the State Ambulatory Surgery Databases (SASD). Available at: www.hcup-us.ahrq.gov/sasdoverview.jsp. Accessed October 23, 2013.

[13] Jacobs WC, van Tulder M, Arts M, Rubinstein SM, van Middelkoop M, Ostelo R, Verhagen A, Koes B, Peul WC (2010). Surgery versus conservative management of sciatica due to a lumbar herniated disc: a systematic review. Eur Spine J..

[14] Malter AD, Larson EB, Urban N, Deyo RA (1996). Cost-effectiveness of lumbar discectomy for the treatment of herniated intervertebral disc. Spine Phila Pa.

[15] Medicare Payment Advisory Committee (Medpac). Report to the Congress: Medicare Payment Policy. March 2005. Available at: http://www.medpac.gov/documents/Mar05_entirereport.pdf. Accessed December 16, 2013,

[16] Osterman H, Sund R, Seitsalo S, Keskimäki I (2003). Risk of multiple reoperations after lumbar discectomy: a population-based study. Spine (Phila Pa 1976).

[17] Papadopoulos EC, Girardi FP, Sandhu HS, Sama AA, Parvataneni HK, O’Leary PF, Cammisa FP (2006). Outcome of revision discectomies following recurrent lumbar disc herniation. Spine Phila Pa.

[18] Peul WC, van Houweilingen HC, van den Hout WB, Brand R, Eekhof JA, Tans JT, Thomeer RT, Koes BW (2007). Leiden-The Hague Spine Intervention Prognostic Study Group. Surgery versus prolonged conservative treatment for sciatica. N Engl J Med..

[19] Ruiz D, Koenig L, Dall TM, Gallo P, Narzikul A, Parvizi J, Tongue J (2013). The direct and indirect costs to society of treatment for end-stage knee osteoarthritis. J Bone Joint Surg Am..

[20] Stewart WF, Ricci JA, Chee E, Morganstein D, Lipton R (2003). Lost productive time and cost due to common pain conditions in the US workforce. JAMA..

[21] Suk KS, Lee HM, Moon SH, Kim NH (2001). Recurrent lumbar disc herniation: results of operative management. Spine (Phila Pa 1976).

[22] Tosteson AN, Skinner JS, Tosteson TD, Lurie JD, Andersson GB, Berven S, Grove MR, Hanscom B, Blood EA, Weinstein JN (2008). The cost effectiveness of surgical versus nonoperative treatment for lumbar disc herniation over two years: evidence from the Spine Patient Outcomes Research Trial (SPORT). Spine (Phila Pa 1976).

[23] Tosteson AN, Tosteson TD, Lurie JD, Abdu W, Herkowitz H, Andersson G, Albert T, Bridwell K, Zhao W, Grove MR, Weinstein MC, Weinstein JN (2011). Comparative effectiveness evidence from the spine patient outcomes research trial: surgical versus nonoperative care for spinal stenosis, degenerative spondylolisthesis, and intervertebral disc herniation. Spine (Phila Pa 1976).

[24] United States Bone and Joint Initiative (2011). The Burden of Musculoskeletal Diseases in the United States.

[25] Veresciagina K, Spakauskas B, Ambrozaitis KV (2010). Clinical outcomes of patients with lumbar disc herniation, selected for one-level open-discectomy and microdiscectomy. Eur Spine J..

[26] Weinstein JN, Lurie JD, Tosteson TD, Skinner JS, Hanscom B, Tosteson AN, Herkowitz H, Fischgrund J, Cammisa FP, Albert T, Deyo RA (2006). Surgical versus nonoperative treatment for lumbar disk herniation: the Spine Patient Outcomes Research Trial (SPORT) observational cohort. JAMA..

[27] Weinstein JN, Lurie JD, Tosteson TD, Tosteson AN, Blood EA, Abdu WA, Herkowitz H, Hilibrand A, Albert T, Fischgrund J (2008). Surgical versus nonoperative treatment for lumbar disc herniation: four-year results for the Spine Patient Outcomes Research Trial (SPORT). Spine (Phila Pa 1976).

[28] Weinstein JN, Tosteson TD, Lurie JD, Tosteson AN, Hanscom B, Skinner JS, Abdu WA, Hilibrand AS, Boden SD, Deyo RA (2006). Surgical vs nonoperative treatment for lumbar disk herniation: the Spine Patient Outcomes Research Trial (SPORT): a randomized trial. JAMA..

[29] Work Loss Data Institute. *Official Disability Guidelines: Special Edition Top 200 Conditions.* 17th ed. Encinitas, CA: Work Loss Data Institute; 2012.

